# Agar Sediment Test for Assessing the Suitability of Organic Waste Streams for Recovering Nutrients by the Aquatic Worm *Lumbriculus variegatus*

**DOI:** 10.1371/journal.pone.0149165

**Published:** 2016-03-03

**Authors:** Bob Laarhoven, H. J. H. Elissen, H. Temmink, C. J. N. Buisman

**Affiliations:** 1 Sub-department of Environmental Technology, Wageningen University, P.O. Box 17, 6700 AA, Wageningen, the Netherlands; 2 ACRRES, Wageningen University and Research Centre, P.O. Box 430, 8200 AK, Lelystad, the Netherlands; 3 Wetsus, Centre of excellence for Sustainable Water Technology, P.O. Box 1113, 8911 MA, Leeuwarden, the Netherlands; Northwest Fisheries Science Center, NOAA Fisheries, UNITED STATES

## Abstract

An agar sediment test was developed to evaluate the suitability of organic waste streams from the food industry for recovering nutrients by the aquatic worm *Lumbriculus variegatus* (Lv). The effects of agar gel, sand, and food quantities in the sediment test on worm growth, reproduction, and water quality were studied. Agar gel addition ameliorated growth conditions by reducing food hydrolysis and altering sediment structure. Best results for combined reproduction and growth were obtained with 0.6% agar-gel (20 ml), 10 g. fine sand, 40 g. coarse sand, and 105 mg fish food (Tetramin). With agar gel, ingestion and growth is more the result of addition of food in its original quality. Final tests with secondary potato starch sludge and wheat bran demonstrated that this test is appropriate for the comparison of solid feedstuffs and suspended organic waste streams. This test method is expected to be suitable for organic waste studies using other sediment dwelling invertebrates.

## Introduction

The freshwater worm *Lumbriculus variegatus* (Lv) (Oligochaeta, Lumbriculidae) is a natural fish food and can be employed for the reduction and compaction of municipal wastewater sludge. However, application of produced worm biomass, as livestock feed poses a significant risk due to the presence of heavy metals, micro pollutants, and pathogens [1,2]. Worm biomass, therefore, should be produced from safe waste streams such as byproducts from food industries. To evaluate worm growth performance on different organic waste streams, the growth and reproduction of Lv on these streams should be quantified in relation to a standard food substrate. This paper describes the development of an standardized agar sediment test for this purpose.

The characteristics described below show that Lv is a promising species for recovering high value nutrients (particularly protein) from food byproducts to produce worm biomass for application as a high-quality commercial feed source; in particular, as aquaculture feed [3,4]

Lv has a gross nutritional value similar to other fish diets such as brine shrimp and trout chow and satisfies fish nutrition guidelines with respect to proteins and essential amino acids [5]. Lv dry matter (14–16% of the live weight) contains 62–66% protein, 11–12% lipids, 4–9% ash and 7–12% fatty acids [2,6]. Reproduction of Lv occurs by simple year-round division (architomy), and worm populations can double their biomass every two weeks [7]. They also possess a high tolerance for temperature fluctuations with an optimum between 15 and 25°C and optimum oxygen levels are between 2.3 and 8.1 mg O_2_/L [8,9]. It has not been identified which range of food sources Lv is capable of utilizing, although its nutritional requirements probably do not vary from other sediment-feeding invertebrates such as macro benthos which feed on complex mixtures of decomposing organic matter, algae, and microorganisms [10,11]. Accordingly, in nature, Lv dominantly ingests subsurface sediments containing the digestible organic fraction which is associated with this sediment [12]. In many laboratory cultures, Lv feeds on fish food such as Tetramin® whereas, for sludge treatment, it feeds on a complex mixture of biopolymers and bacteria[1].

Food type should be the only variable when comparing different food sources as a substrate for Lv production. Such a test should comprise ideal food uptake conditions with limited food hydrolysis combined with a fixed sediment composition and structure as well as the continuous refreshment of overlying water [13]. For the development of a standardized growth test for assessing a variation of potential food sources, it is essential to understand that many factors have a(n) (in)direct impact on worm growth and reproduction. This includes sediment type (particle size, organic fraction); food source (concentration, composition, particle size); and (pore) water quality [8,13,14,15,16,17].

Laboratory tests employing Lv have been developed especially for toxicology and bioaccumulation studies [18] and activated sludge reduction [19]. However, for testing food byproducts, these tests are likely to prove ineffective as water quality (e.g. ammonia and oxygen levels) will be negatively affected by their degradation products. Also standard sediment-bioaccumulation tests specify that Lv should not be fed during the 28-day exposure [20] in order to maintain water quality and to avoid any disturbance in contaminant bioavailability.

For inhibiting the transfer of soluble food compounds from the sediment into the overlying water and to promote direct feeding, we propose utilizing agar gel to trap food components inside the sediment. This is similar to the application of agar in microbial cultures where the agar gel is used as a fixed gel medium and often mixed with nutrients essential for growth. Agar is also able to replace soil and water in laboratory tests for *Enchytraeidae* (potworms) [21].

Agar can be consumed by the earthworm *Perionyx excavatus*, substantiating that direct uptake occurs [22]. Under normal experimental conditions, it is unlikely to be digested by Oligochaeta as the polymer only becomes unstable when heated above 90°C and exposed to a pH exceeding the range of 5.5–8. Degradation by agarases can be neglected as these are normally not present in food products not related to marine environments or active under freshwater conditions [23].

The objective of this study was to develop a laboratory test procedure for a reliable comparison of growth and reproduction of Lv on a variety of food sources. TetraMin® fish food was exploited as a food by-product model during the test development since it contains a balanced mixture of nutrients that dissolve and break down over a period of time, suitable for long-term worm culture. Finally, secondary potato starch sludge and wheat bran, were employed in the test as examples of real food byproducts, to ensure its practical applicability, substantiating that this new agar-based test is a reliable method for evaluating the suitability of different food byproducts for producing worm biomass.

## Materials and Methods

### Experimental design

Experiments 1, 2, and 3 were performed with different combinations of agar, sand and TetraMin® as food (Table 1) in order to select a test setup most suitable for growth and reproduction of Lv over a 21 day timeframe. A final test of 25 days (Experiment 4) with Tetramin, secondary potato starch sludge (starch production, Novidon, the Netherlands) and wheat bran (Cargill, the Netherlands) was performed to study growth, reproduction and suitability of the selected test setup for further use with a genuine food byproduct. In the selected agar test setup, an oxygen profile was determined and a food hydrolysis test was conducted to examine the effect of agar on oxygen concentrations and ammonium production.

**Table 1:** The effect of specific combinations of agar gel, sand, and food on final worm numbers and changes in total wet weight (%).

		Agar		Sand		Food	Final worm	
Exp.	Test	Conc.(%)	Amount	fraction	Amount	Amount	Number	Weight (%)
**1**	1	0	0	C	90	0	99	-16,1
** **	2	0,8	30	none	0	0	56	-14,4
** **	3	0,8	30	C	90	0	92	2,5
** **	4	0,8	30	C	90	420	51.0 (3.0)	-13.6 (3.6)
** **	5	0,8	30	C	90	840	47.5 (1.5)	-16.1 (7.5)
** **	6	1	30	C	90	420	55.5 (2.5)	3.7 (2.1)
** **	7	1	30	C	90	840	50.5 (0.5)	-4.1 (1.4)
**2**	1	0	0	C	90	420	67	9,9
** **	2	0	0	C	90	1050	46	-12,2
** **	3	0	0	F/C	22.5/67.5	420	62	9,1
** **	4	0	0	F/C	22.5/67.5	1050	50	3,3
** **	5	0,6	30	C	90	420	65	41,3
** **	6	0,6	30	C	90	1050	54	7,5
** **	7	0,6	30	F/C	22.5/67.6	420	59	40,6
** **	8	0,6	30	F/C	22.5/67.6	1050	52	11,1
** **	9	0,8	30	C	90	420	64	22,8
** **	10	0,8	30	C	90	1050	53	8,2
** **	11	0,8	30	F/C	22.5/67.7	420	76	44,5
** **	12	0,8	30	F/C	22.5/67.7	1050	54	19,1
**3**	1	0	0	F/C	10/40	0	104	-20,6
** **	2	0	0	F/C	10/40	105	56	3,3
** **	3	0	0	F/C	10/40	140	76	5,2
** **	4	0	0	F/C	10/40	210	50	-6,1
** **	5	0,6	20	F/C	10/40	0	97	-8,8
** **	6	0,6	20	F/C	10/40	105	79	38,3
** **	7	0,6	20	F/C	10/40	140	52	28,7
** **	8	0,6	20	F/C	10/40	210	50	-10,8
** **	9	0,8	20	F/C	10/40	0	^a^	^a^
** **	10	0,8	20	F/C	10/40	105	64	22,1
** **	11	0,8	20	F/C	10/40	140	61	29,4
** **	12	0,8	20	F/C	10/40	210	50	17,5

Concentrations (Conc.) expressed as weight percentage, amounts of agar gel and sand in grams, and food in milligrams. Sand fractions used: coarse sand (C) and fine sand (F). Test 4–7 measured in duplicate; range is given between brackets (±). Initial worm number = 50 (all tests). Average total start weight Exp.1: 365 mg (min-max, 362–367), Exp. 2: 399 mg (min-max, 330–476), Exp.3: 391 mg (min-max, 350–422). Exp.3

^a^Test 9: Worm losses due to experimental error

The duplicates in Experiment 1 confirmed that the maximum standard deviation within one single test combination was at most 7.5%. Therefore further combinations tested in experiments 2 and 3 were not tested in duplicate to increase the number of tested combinations. In addition, the final experiment was done in duplicate, to further validate the repeatability in growth and reproduction. Based on the results of Experiment 1, pretreatment of the agar gel and sand addition was improved for Experiments 2 and 3, and the water renewal rate was increased. Additional details are indicated in the information described below.

### Test organisms

*L*.*variegatus* from a recirculating aquaculture system was retained in 16.0 L plastic flow-through tanks. Culture tanks were each fitted with two 1.5 cm thick PVC mats. These mats had an open structure which provided sufficient support for 300 to 500 g of live worms per tank. The cultures were nourished three times per week with fish food flakes (TetraMin® or trout food). Water temperature was maintained at 19°C and filtered over a nitrifying filter to prevent ammonia and nitrite levels above 0.4 mg/L. Prior to entering the tanks, the water was aerated to ensure oxygen levels above 6 mg/L in the tanks. Water refreshment rate was approximately 2.5 h^-1^. Approximately every second week, 30% of the water was replaced by tap water. Fully developed worms from cultures experiencing positive growth and no indications of recent fragmentation (front and hind parts without thickening or blunt ends) or abnormalities were selected and purged overnight in clean tap water to remove their remaining gut content. At the beginning of Experiments 1–4, 50 worms were selected for each test, and their total wet weight was determined (0.3–0.4 g per test).

### Test beaker and system configuration

A controlled temperature and water flow system with 12 plastic flow-through beakers was utilized to test the different agar/sand/food combinations (Table 1). Each beaker had a working volume of 770±8 mL and a bottom surface area of 57 cm^2^. Water was continuously discharged with a drain pipe positioned approximately 2 cm above the artificial sediment (at the 150 mL mark). The beakers were submerged in a temperature controlled bath at 19±1°C and retained in dark conditions until sediment replacement and/or sampling occurred. Water was supplemented at a flow rate of 2.17 ml/min in Experiment 1 and 4.34 ml/min in Experiment 2–4, resulting in refreshment rates of 4 and 8 day^-1^, respectively. The water (pH 8.3–8.5, hardness 92 mg CaCO_3_) was a blend of tap water (Leeuwarden, the Netherlands) and softened water. The water was disinfected with UV-C light to prevent inoculation of the beakers with microorganisms. It was then supplied on the surface of each beaker by a needle dispenser, creating a constant flow of small droplets.

### Sand, agar medium and food use

Two different quartz gravels were utilized. Pre-treated acid washed sand (ALFA (AESAR)) with particle size 0.1–0.35 mm obtained from VWR International (the Netherlands) will be referred to as fine sand (F). Quartz filter sand with particle size 0.71–1.25 mm will be further referred to as coarse sand (C). It was obtained from Wildkamp® (the Netherlands), rinsed with hot water, and combusted at 550°C for 2 hours to eliminate organic compounds. Various agar concentrations were pre-prepared by mixing Agar (BD Bacto™ Agar) with demineralized water, after which the mixture was autoclaved at 120°C for 20 minutes to dissolve the agar. Prior to use, Tetramin flakes were finely ground to create the food particles suitable for unmediated worm ingestion. Yeast extract BBL (BBL 211929) was added as a source of minerals and vitamins. In Experiment 1, all food enriched beakers contained 15 mg (dry weight) yeast extract each; in Experiment 2–4, 20 mg yeast was added for each gram of food.

In Experiment 1, 90 g coarse sand was employed with or without agar gel (Table 1). For beakers containing an agar layer, the gel (30 g) was first poured into the test beakers and cooled before sand was added on top. For each combination with food (0, 420 and 840 mg), the food was incorporated into the agar and water mixture prior to autoclaving.

**Fig 1:**
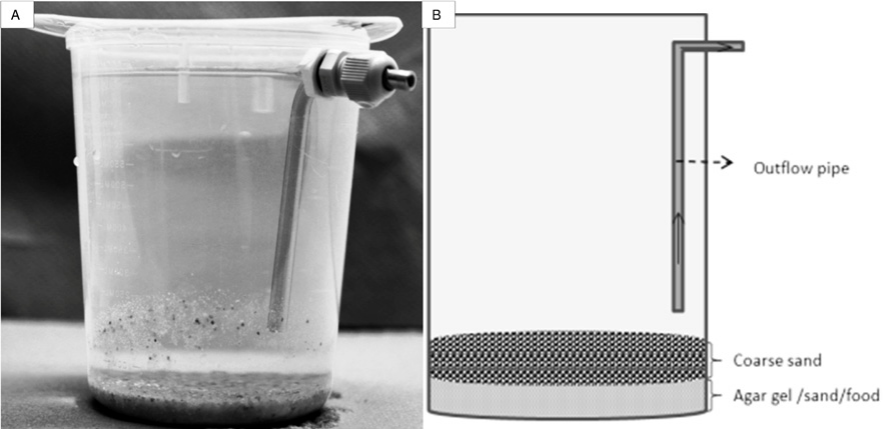
(a) Picture of experimental beaker including artificial sediment. (b) Schematic overview of the artificial sediment structure used in Experiments 2, 3 and 4.

In Experiment 2, the agar gel was treated differently than in Experiment 1. After autoclaving, the gel was cooled and briefly blended to break up the tension of the gel and make it less viscous. Subsequently, 30 g of pre-blended agar gel was mixed with the food (420 and 1050 mg) and part of the sand. Both coarse and fine sand were utilized. The first mixture contained only 90 g coarse sand (C/90; Table 1) which was applied by mixing 45 g sand with food and/or agar gel and adding 45 g sand on top of this mixture. The second mixture consisted of a total of 22.5 g fine and 67.5 g coarse sand (F/22.5 + C/67.5; Table 1) and was prepared by initially mixing 22.5 g fine and 22.5 g coarse sand with food and/or agar gel. This created an artificial sediment layer wherein interstitial space was filled with agar gel and 45 g coarse sand was subsequently added on top (Fig 1).

In Experiment 3, only one mixture of sand was used, and the total amount of sand was reduced to 50 g: 10 g of fine sand and 10 g of coarse sand were mixed with only food (0,105,140 or 210 mg), 20 g agar gel, or both. As a final step, 30 g course sand was added on top. The same pre-blended agar gel of Experiment 2 was employed in Experiment 3.

In Experiment 4, the same combination and settings utilized in Test 6 (Exp. 3) were used with three different diets all tested in duplicate. To standardize the organic loading between the three applied diets, chemical oxygen demand (COD) and total nitrogen were measured according to Standard Methods[24] using Hach Lange® test kits. Tetramin, secondary potato starch sludge and wheat bran respectively contain 1.33, 1.25 g and 1.07 COD/g dry weight and 0.075, 0.059 and 0.023 g total nitrogen/g dry weight. For both, an amount representing 140 mg COD was added comprising 105 mg and 131 mg for Tetramin and wheat bran respectively and 6 to 10 ml of fresh sludge. The diets were tested over 25 days starting with 50 worms each. The entire sediments including the food substrate were replaced three times per week. Wheat bran was tested at a later stage using worms from a different batch with a higher individual weight.

### Water quality measurements

Once per week, dissolved oxygen (DO) concentrations and pH were measured *in situ* prior to artificial sediment replacement using a Hach® Luminescent Dissolved Oxygen probe (LDO) and a pH electrode (WTW®, Sentix® Plus). To ensure precise and stable measurements at 0–2 mm above the sediment surface, these probes were inserted into the test beaker with a custom-made probe holder. Once per week, DO and pH concentrations were also measured in the incoming water, and total ammonium nitrogen (TAN) concentration was determined with Hach Lange® test kits (HACH LANGE LCK304). For this purpose, samples were extracted at 0–2 mm above the surface of the sediment with a 5 ml syringe and needle.

### Investigation of food hydrolysis and oxygen profile in optimized test setup

To examine the differences in breakdown of Tetramin under test conditions with and without agar gel, 420 mg of food was incorporated into 80 ml of water or agar gel (0.6% w/w) which corresponds to the additions in the optimized beaker setup. Both mixtures were sealed in a 100 ml syringe, depleted of remaining air, and incubated for two days at a temperature of 19°C. Finally, a sample was withdrawn from each syringe, diluted, and analysed for TAN.

In order to study the DO levels within and above the sediment in more detail, an oxygen micro sensor (PreSense®, Sensor type PSt1) was used. Two beakers with an optimized test setup similar to Test 6 (Exp. 3) and containing 50 worms each were used. The test beakers were removed from the temperature controlled bath prior to measurement. Measurement began when the sensor was slowly inserted in the sediment and gradually moved upwards in moderate increments varying between 0.4–2 mm. Sensor position was determined and controlled with the use of a digital caliper which was connected to the sensor probe. The positions of the caliper and sensor probe were stabilized by a solid aluminum frame surrounding the test beaker to minimize vibrations during repositioning of the sensor.

### Replacement of artificial sediment and worm handling

To minimize bacterial growth and associated food degradation, complete sediments including the food were replaced three times per week in all experiments. The test beakers were withdrawn from the temperature controlled bath, and approximately 70% (v/v) of the overlying water was removed. The beaker was gently shaken to fragment the sediment layer which incited most worms to perform their typical escape reflex [25] into the overlying water. This reflex made it possible to collect the worms by pouring off the overlying water. Repeating this three times resulted in complete removal of the worms from the sediment. The collected worms were cleaned by flushing them with clean water, and all of the sediments were thoroughly inspected for remaining worms. The worms were placed directly into clean test beakers with new sediments and 200 ml of water. Once per week, total worm wet weight was measured with a pre weighted fine mesh, and total number of worms were determined by counting the worms using a glass pipette. Prior to weighing, worms were collected on top of the mesh, and paper towels were gently pressed against the back of the mesh for 10 seconds to absorb adherent water.

## Results and Discussion

### Experiment 1: single and combined effects of agar and sand

In this experiment, it was expected that the absence of food would result in a reduction of total weight and in a consistent number of worms. When food was included in the agar gel, increases in total weight and worm numbers were expected. In all of the tests with agar, it was observed that the worms were not capable of penetrating the agar layer. The absence of faecal pellets and reductions in total weight between 4.1 and 16.1% in Tests 4, 5 and 7 (Table 1) demonstrate that food uptake was probably limited for all combinations containing food and agar gel.

**Fig 2:**
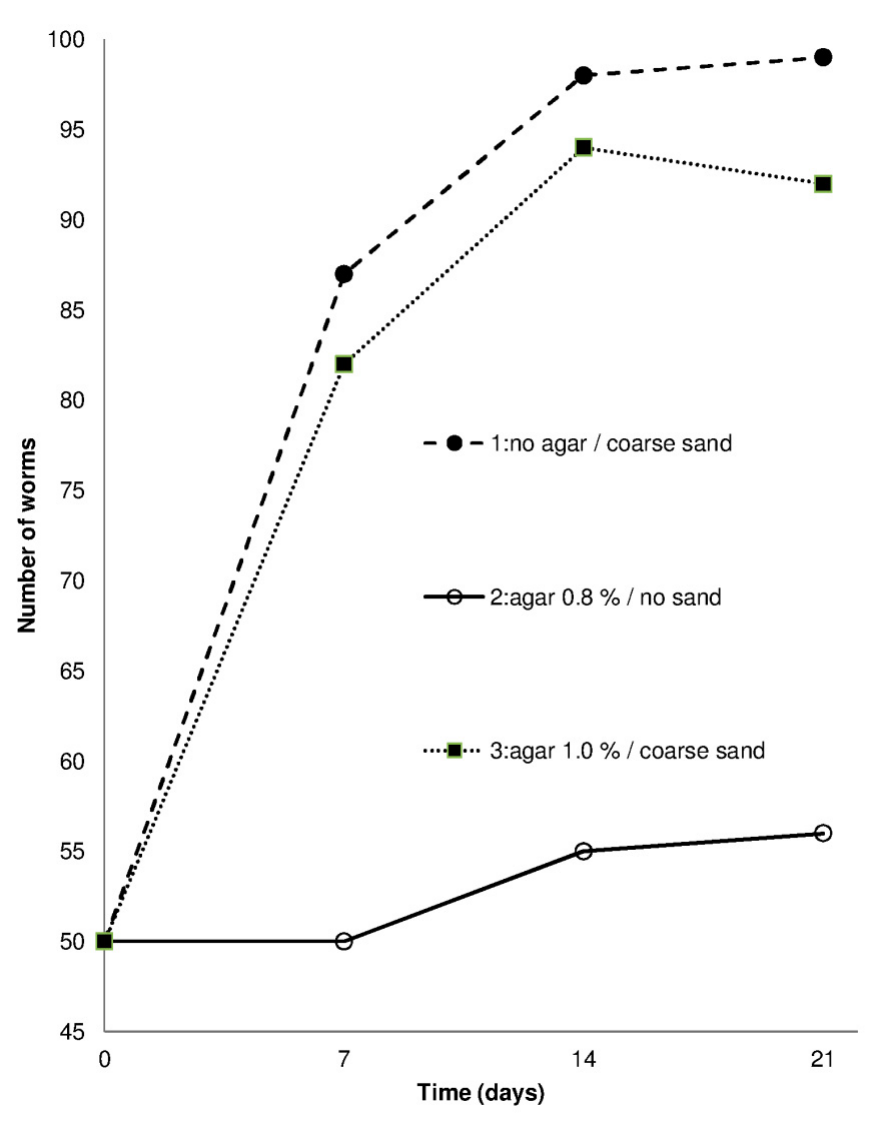
Experiment 1: Change in worm numbers during 21 days for three test combinations without food. For agar gel and coarse sand 30 g and 90 g were used, respectively.

Tests 1 and 3, characterized by the absence of food in combination with coarse sand or a combination of agar and coarse sand, unexpectedly resulted in a more significant increase in worm numbers than in Test 2 with the omission of sand (Fig 2). The major portion of this increase occurred during the first week of the test, and the worm weight was reduced considerably in test1, 2 and was negligible in Tests 3 (Table 1).

Test 6, with a combination of 1.0% agar gel, 90 g coarse sand, and 420 mg food only exhibited a moderate weight increase between 2% and 6%. With 50 worms initially, the final worm numbers in all of the tests that included food varied between 46 and 58 worms due to mortality and reproduction, respectively.

Within two days from the beginning of the tests, all beakers containing food displayed an opaque stagnant layer above the sediment with elevated TAN levels, i.e. tests with 420 mg food and 840 mg food exhibited concentrations of 0.6–1.0 and 1.1–2.2 mg NH_4_^+^-N /L, respectively.

In a control beaker without worms but including 30 ml of 1.0% agar, 90 g. of coarse sand, and 840 mg of food, levels were in the same range as in the other beakers with 840 mg food and worms (1.6–1.7 mg NH_4_^+^-N /L), demonstrating that ammonium release is primarily related to the added food amount in the test and is less influenced by worm presence. In addition, all of the tests that included food exhibited a decrease in DO from ± 7.6 to lower than 1 mg/l O_2_ close to the sediment, and a pH decrease from 8.3 to approximately 7.4.

In Experiment 1, no suitable combinations were found that promoted both worm growth and reproduction. Elevated TAN levels and an emergence of an opaque stagnant layer above the sediment indicated that heat pretreatment of the food had stimulated undesired food hydrolysis. Changes were made to improve the accessibility and consumption of agar and the food particles encapsulated by the agar. In Experiment 2, it was decided to employ only 0.6 and 0.8% agar-gels which were stirred and shaken after solidification to lower the viscosity and surface tension. To minimize the dissolution and decomposition of food due to elevated temperature exposure, food particles were mixed with the agar-gel only after it was cooled and stirred, creating a suspension of food particles in the agar-gel.

In addition, to stimulate the worms’ consumption of small particles, fine sand was added and mixed with coarse sand and agar-gel. Based on algae consumption data, it was ascertained that Lv is capable of consuming particles up to 200 μm [26]. As there were no clear variances in Experiment 1 in total wet weight or worm numbers between tests with 420–840 mg of food, it was also decided to apply a broader range of food amounts. The water renewal rate was doubled from 4 to 8 day^-1^ to remove more ammonium and provide additional oxygen.

### Experiment 2: the effect of agar gel, sand and two different food levels

**Fig 3:**
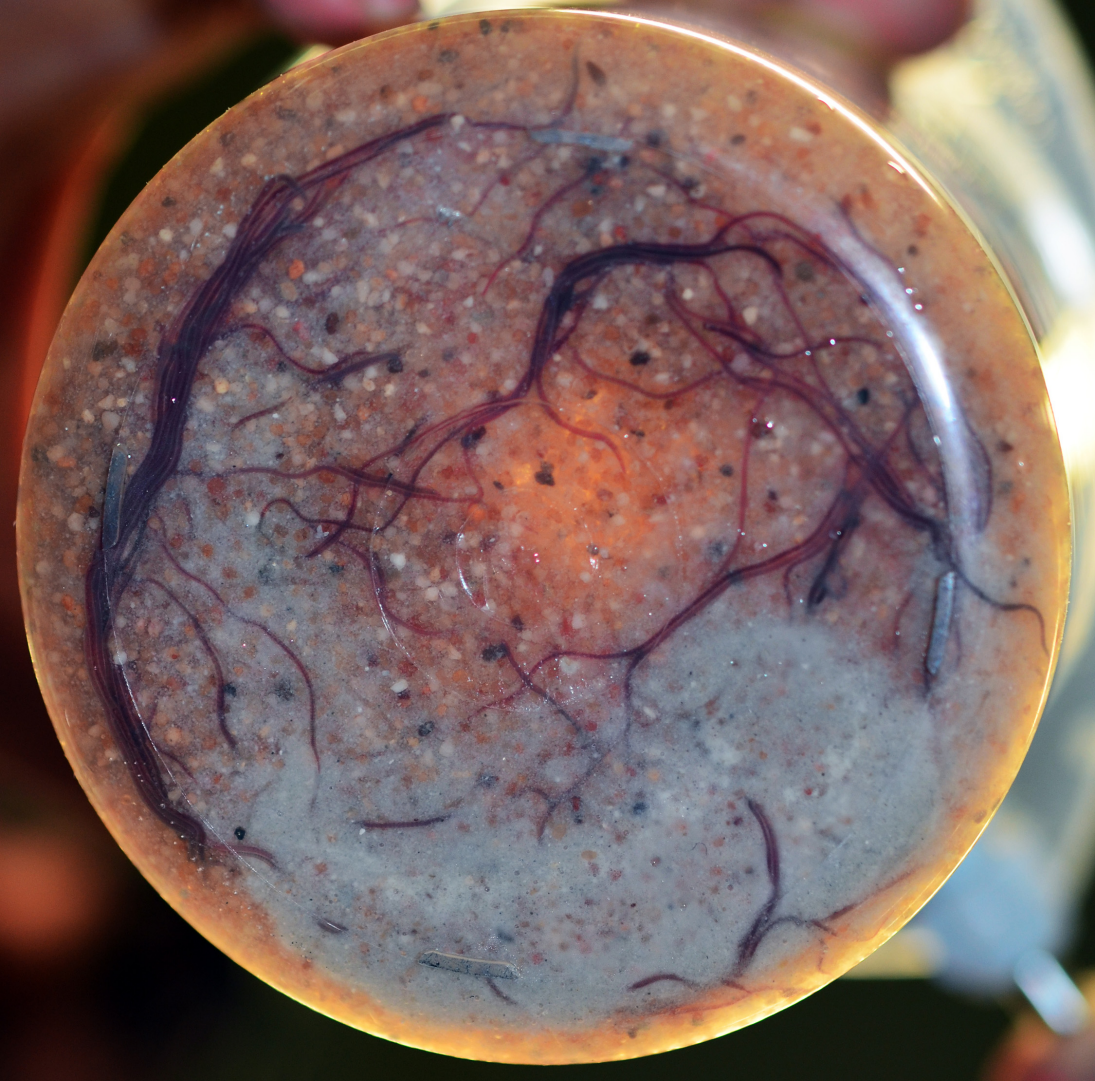
Bottom view of test beaker containing an agar layer combined with a mixture of sand and food which is penetrated by worms.

In all test beakers in which this new agar gel pretreatment was applied, worms were able to penetrate the entire sediment (Fig 3). A microscopic examination of the deposits on the top of the coarse sand layer revealed that faecal pellets were produced containing gel and fine sand confirming that active consumption had occurred. Growth and reproduction were found for all of the combinations with agar.

All combinations containing agar gel demonstrated more increase in total weight than combinations without agar. No variance was observed between the 0.6% and 0.8% agar gel (Table 1). Differences in the total weight increase and number of worms were primarily associated with the food amount and the addition of agar gel but less with the use of different sand types or agar gel concentrations. Increase in worm weight and numbers were determined at low food amounts as can be observed when comparing tests with 420 mg opposed to tests with 1050 mg of food. With the latter food level, a positive trend in total weight was observed for the coarse-fine sand type and greater agar concentrations.

The effects of agar, sand and food on DO and pH (S1 Fig) were not evident due to significant variations in these measurements. An overall trend of lower TAN levels in tests with agar gel was observed compared to tests without agar. Tests with elevated food amounts exhibited higher TAN levels than tests with low food amounts. The sand type and agar concentration only seemed to play a minor role. Elevated TAN levels again correlated with an opaque layer just above the sediment surface in the beakers wherein agar gel was omitted (Test 1–4), exhibiting a thicker opaque layer. With an addition of agar gel, thin or no distinct opaque layers were formed or were even nonexistent in beakers with less food (420 mg), revealing that this is associated with the amount of added food. A pH decrease within all test beakers was ascertained similar to Experiment 1. It appears that pH diminishes more acutely with the absence of agar gel, but this could not be further established.

Lower TAN levels in tests with gels were plausibly caused by less food degradation. Despite doubling of the water renewal rates, DO levels above the sediment surface remained very low compared to the levels in Experiment 1.

### Experiment 3: Different agar gel concentrations combined with lower food levels

In Experiment 3, the effects of agar addition and concentration (0.6 and 0.8%) were studied with lower food levels of 0, 105, 140, and 210 mg which were intended to reduce the negative effect on reproduction and growth that had been found at the higher food levels in Experiment 2. Several other changes were made to improve the conditions for feed uptake: 1) a thinner coarse sand layer to increase access to the layer of food containing gel; 2) a decrease in the gel to food ratio (w/w); and 3) the use of a fixed mixture of coarse and fine sand.

Reproduction in the absence of food (Test 1 and 5) was elevated and accompanied by a reduction of the total weight (as previously observed in Tests 1 and 3, Exp. 1). Tests with food demonstrated no reproduction at the highest food level of 210 mg (Tests 4, 8, and 12) in combination with negative growth in Tests 4 and 8 and positive growth in Test 12. A low to moderate reproduction response was ascertained at the 105 and 140 mg food levels compared to tests without food. At the two lowest food levels, a more than 22.1% growth (in 21 days) was determined in the presence of agar gel. No obvious effects of agar gel or its concentration on reproduction could be determined. However, visual observation established that the addition of agar gel results in an improved worm distribution as well as improved conditions for food uptake as the worms penetrated the entire sediment layer.

The effects of agar and food on TAN, DO and pH were remarkably variable. Nevertheless, TAN levels increased with the amount of food and were reduced in the presence of agar gel. The lowest TAN levels were observed in the absence of food.

In all of the test beakers containing food, pH decreased from an average inflow pH of 8.4 (SD, 0.14) to values below 7.4 near the sediment. In the test without food (Tests 1, 5 and 9), the pH decrease was more moderate. In general, higher levels of food lead to a greater decline in pH and a more clearly defined opaque layer above the surface of the sediment.

### Effect of sand on reproduction and growth

Sand stimulates worms to reproduce (Experiments 1 and 3). However, Experiment 3 demonstrated that combining sand with food initially reduced reproduction. Experiment 2 showed that a mixture of fine and coarse sand induced up to 10% more worm weight with the greatest food addition than in coarse sand alone. Apparently, fine sand has a positive effect on growth at elevated food levels. However, reproduction did not vary between the two sand/sediment types. The effect of sand on growth and reproduction of Lv is not yet fully understood. An additional study indicated that reproduction of Lv in natural sediments is lower in sandy sediments with low organic carbon [27]. In the same study, reproduction was determined to be worm size dependent with larger worms reproducing more frequently. It is believed that, in our tests with only sand, reproduction was the result of the absence of organics which may have incited a stress induced reproduction response. In the beakers with food, reproduction was often delayed, indicating that the worms must achieve a certain size before reproduction occurred. This is in accordance with observations that specific culture conditions will determine at what size the animal will reproduce during the test [14]. Factors mentioned to be of influence on the minimum size for reproduction are food availability, food quality, and water quality. The ingestion of sand by Lv has been substantiated to be significant for the uptake of hydrophobic compounds [12]. Therefore, a certain minimum amount of fine sand that is small enough for ingestion should be added to increase the bioavailability of hydrophobic compounds.

Standardized invertebrate bioassays are generally initiated with animals of a defined age- or stage-structure to control for individual variability in the measured response [28]. As the reproductive strategy of Lv creates significant difficulty for age or stage selection, the directly induced reproduction in sediments without food that was observed in our experiments could also be used prior to testing to improve overall standardization in bioassays.

### Experiment 4: Validation of optimized test setup

**Fig 4:**
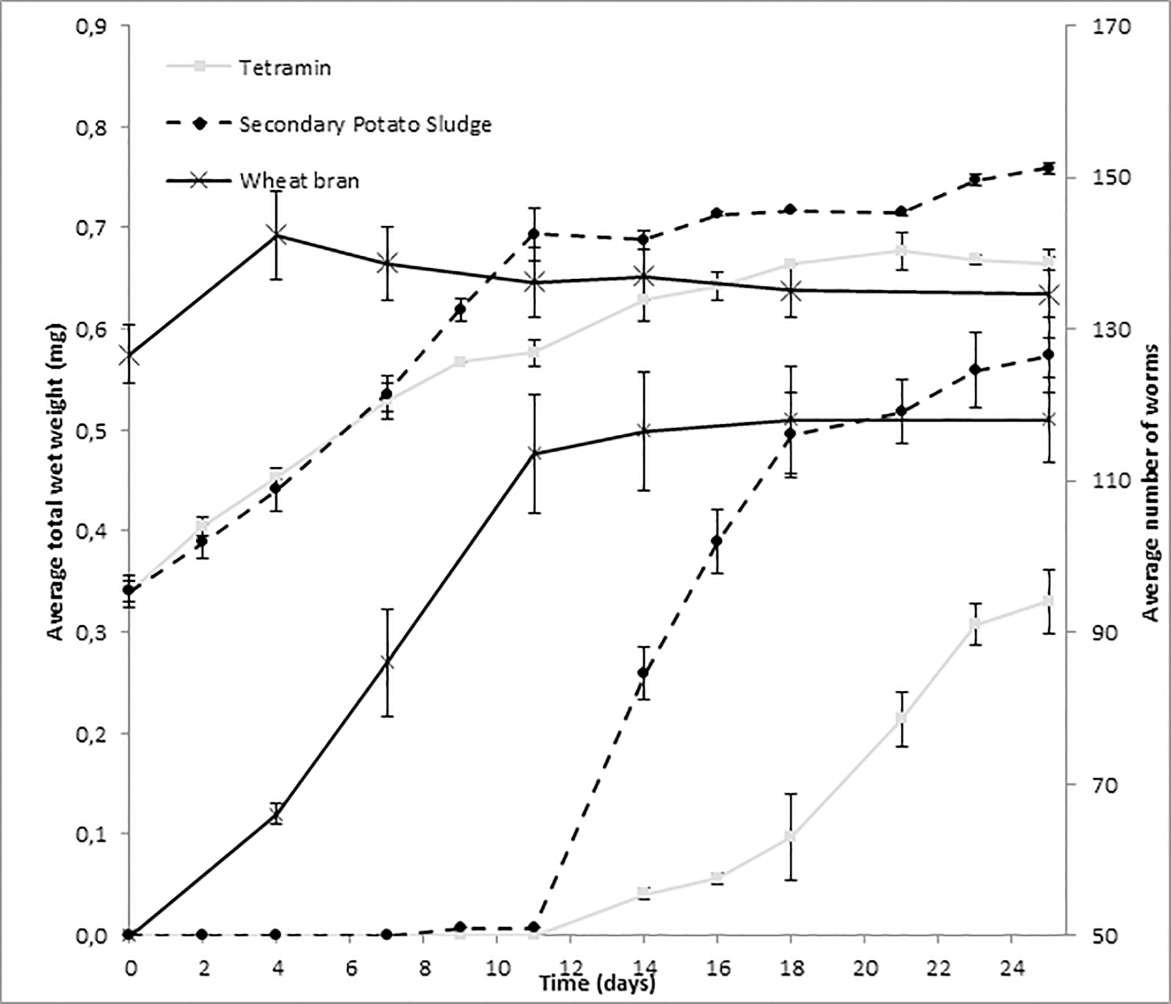
Increase in *L*. *variegatus* live weight and number in an optimized growth setup. Setup contains 0.6% agar-gel (20 ml), 10 g. fine sand, 40 g. coarse sand and 140 mg COD for three different diets (Tetramin, secondary potato sludge and wheat bran). Numbers of worms (right axis) are represented by the three lowest lines and increase in total wet weight (left axis) by the three upper lines. Error bars; average of two biological replicates ± standard deviation.

A test combination of 0.6% agar-gel (20 ml), 10 g fine sand, 40 g coarse sand and 105 mg Tetramin, combined with a water renewal rate of 8 day^-1^ was most effective and used for further experiments. Fig 4 shows that both Tetramin and secondary potato starch sludge diets supported reproduction and growth for the entire test period. The graph depicts that reproduction begins on day 11 for both diets. The total wet weight increase was initially higher due to individual growth in the absence of reproduction. After day 11, the overall growth rate decreased with the initiation of reproduction. Potato sludge supported a higher overall growth rate and reproduction than Tetramin. In contrast to Tetramin and secondary potato starch sludge diets, worms cultivated on wheat bran showed a small increase in total weight and a reproduction that almost stopped after day 11. This test that started with a larger initial weight and a nitrogen poor diet showed that both initial weight and the quality of the diet can influence the overall response.

Standard deviation within the tested diets never exceeded 7.0% for total wet weight and 8.9% for the number of worms. TAN, DO, and pH values were similar to those under the selected standard conditions from Test 6 of Experiment 3 (data not shown). The final increase in worm numbers and increase in total weight differed clearly between Tetramin, Potato sludge and wheat bran diets. Increase in worm numbers were respectively 44, 76.5 and 68 and increase in total weight were respectively 0.32, 0.42 and 0.06 g (25 days). Results demonstrate that the diet has a dominant effect on growth rate and reproduction. When sediments are replaced three times per week, this combination of agar, sand, and food was appropriate to monitor growth and reproduction for a test of at least 25 days.

Based on Experiment 3, it is believed that food was not limiting when 105 mg Tetramin was added. To determine the optimum food level for testing, a food saturation growth curve should be established such as those that have been established for early instar chironomid larvae[10]. In that study it was demonstrated that maximum specific growth rates strongly depend on food composition and quality. It is surmised that different food types, food levels, and food renewal times, therefore, will result in different Lv growth rates. It is recommended to add less food in the standard setup to prevent possible ammonium accumulation when a COD/N (w/w) ratio below 18 (Tetramin) is utilized.

Growth rates between 0.02–0.05 d^-1^ were achieved in Tetramin in a previous study investigating the feeding behavior of Lv [29]. Similar growth rates were observed in Experiments 2 and 3 for several combinations of agar, sand, and food. In the selected combination for long term testing (exp. 4), growth rates (t = 0–25) of 0.027 and 0.032 d^-1^ were achieved for Tetramin and secondary potato sludge, respectively. The initial growth rates (t = 0–7 days) in Tetramin and potato sludge were, respectively, 0.063 and 0.065 and are in the same range as the initial growth rates ascertained in waste activated sludge of 0.05–0.11 d^-1^ [19].

In the latest sediment bioaccumulation test with Lv, a maximum wet weight increase of 48% within 28 days (calculated growth rate approximately 0.014) was found [20]. Comparing the growth rates between the sediment bioaccumulation tests and the new agar sediment test showed that growth rate was almost doubled in the latter test (growth rate of 0.027 instead of 0.014). Although the agar-sediment test is different in several aspects (e.g. the use of agar, incubation temperature, photoperiod, sediment composition and replacement) we expect that the large difference in growth rate is dominantly explained by the difference in food dosing. In the agar sediment test 105 mg Tetramin was fed three times a week to a beaker of 770 ml, housing 340 mg of Lv in contrast to only 6 mg Tetramin dosed three times a week to a beaker of 300 ml, housing 250 mg worms in the sediment bioaccumulation test.

### Determination of food hydrolysis and oxygen profile in an agar sediment test setup

The decomposition of Tetramin under test conditions with and without agar was examined. Food decomposition in water was greater than in agar gel, resulting in concentrations of 10 mg/L NH_4_-N and 6 mg/L NH_4_-N, respectively. This reflects a food breakdown of 2.6% (w/w) in water and 1.5% (w/w) in agar gel, indicating that food hydrolysis is diminished when agar-gel is applied. Tests incorporating agar gel in Experiments 2 and 3 generally exhibited lower TAN levels above the sediment, substantiating that protein degradation inside the agar-sediment layer is more gradual than in the absence of agar gel. The agar gel most likely slows down the diffusion of oxygen, resulting in a slower breakdown of the food.

**Fig 5:**
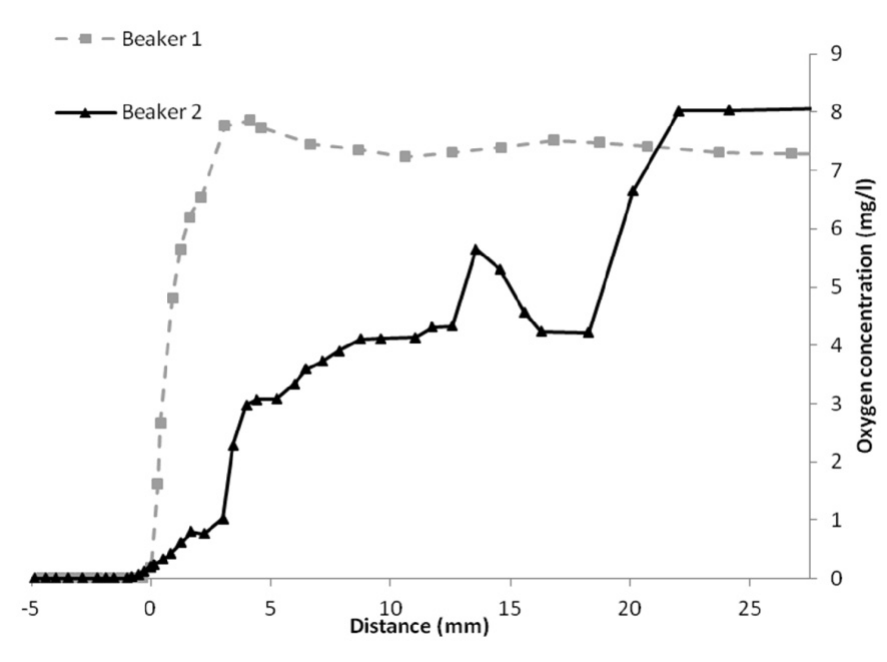
Oxygen profile of two optimized beakers in an identical setup. Both setups contain 0.6% agar-gel (20 ml), 10 g. fine sand, 40 g. coarse sand and 105 mg TM, including 50 worms, incubated for two days prior to measurement. Negative distance refers to measurements in the sediment itself. Transition from sediment to water is indicated by 0 mm; the water outflow is situated around 22 mm distance.

The oxygen levels of two beakers containing worms under conditions identical to Test 6 in Experiment 3 (Fig 5) indicate that, at the interface between the sediment and the overlying water, an intense shift in the oxygen level appears from anoxic to levels up to a maximum of 7 mg/L. However, as it was required to remove the beakers from the controlled temperature and flow system prior to measurement, water mixing occurred, and the gradients demonstrated a significant variation. The oxygen gradient in beaker 2 is, therefore, unlikely to reflect the stable gradient during testing, and it is more plausible that a steep and stable oxygen gradient develops over a period of time such as beaker 1 continued to partially exhibit. The opaque layers in Experiments 2 and 3 support the development of this stable layer near the bottom which, over a period of time, leads to oxygen depletion. To avoid these low oxygen concentrations above the sediment, mixing could be induced by cooling the incoming water to less than the beaker temperature.

Maintaining elevated DO concentrations at the substrate level improves worm growth and survival but also increases endogenous decomposition of the food as demonstrated in activated sludge tests with Lv [19]. A study regarding the effect of DO on Lv fed with waste sludge showed that oxygen concentrations affect food consumption rates [9]. The most significant consumption rates were found at levels above 8.1 mg/L O_2,_ and the lowest food consumption rates were found below 2.5 mg/L O_2_. Constant low DO levels led to a reduction in growth and eventually worm death. In our study, DO levels proximate to the sediment in the food containing beakers were far below 2.5 mg/L with no indications of mortality. This was almost certainly due to the worms having unhindered access to oxygen rich water by protruding their tails above the oxygen depleted layer.

The food hydrolysis test and the oxygen profile establish that food decomposition of the gel enclosed food particles was slowed down by low oxygen levels inside the gel layer. As the sediment layer in the final test setup is oxygen deficient, food decomposition occurs primarily under anaerobic conditions which retards endogenous breakdown as is also found for the decay of activated sludge under anoxic conditions [30].

Assuming that high oxygen levels in the sediment promote sediment dwelling activities of the worms, this is likely to increase the feed intake and thus resulting in a higher growth rate. An elevated DO level in the sediment could at the same time accelerate the breakdown of the food substrate itself resulting in a negative influence on growth and growth conditions. Therefore the influence of higher DO levels in the sediment on growth rate remains unclear and should be further evaluated.

### Effect of agar gel and food amount on worm growth and ammonia

Agar gel addition provided appropriate conditions for growth when combined with a low food level. Worm numbers were not impacted by adding agar gel. TAN concentrations were greater when an overdose of food was added; therefore, food amounts should be retained within a certain range to minimize the effect of ammonia on growth. Toxicity of ammonia in the proposed setup is minimized by keeping ammonia concentrations below 1 mg/l at a pH between 7–8 by applying regular substrate changes, continuous water refreshing, and minimal food additions analogous with 105 mg Tetramin. By maintaining this regime, effects of low ammonia levels on growth are expected to be absent or insignificant, as a former study showed an LC_50_ (10 days) of 93.5 mg/L and 15.1 mg/L, respectively, at pH 7 and 8 [31].

The improved growth conditions for Lv by adding agar gel are thus caused by a reduction in food hydrolysis and, most likely, also due to increasing the interstitial space of the sediment. This makes the sediment easier accessible to the worms for food consumption. In the optimized beaker setup (Test 6, Experiment 3); the total pore volume without agar gel was 9±1 ml. This volume is increased by more than 200% when 20 ml of agar gel is added which results in a greater interstitial volume and, herewith, in lower food and TAN concentrations in sediments with agar gel. Due to this volume expansion, worms can more easily access and penetrate the entire sediment layer. The higher sediment dwelling activity of the worms also intensifies exposure to sediment and agar gel embedded food particles.

### Assessment of organic waste streams for worm culture purposes

How Lv growth performance determined with the agar sediment test can be extrapolated to worm production on a larger scale was not further quantified in this study. It is expected however that this performance is a good indicator whether if a waste stream can be applied for large scale Lv cultivation or not.

Besides food availability and food quality (evaluated in the agar sediment test) the most important factors improving worm growth on a reactor level are: high dissolved oxygen concentrations, low ammonia levels a temperature that should be between 15–25°C [9]. On a reactor level some microbial ingrowth and food breakdown can be expected resulting in slightly changed food qualities that may have a positive or negative effect on biomass production. Thus, the test is suitable to compare different food sources for their suitability for large scale Lv cultivation but not to predict actual biomass production.

### Long term use of the optimized test setup

Under the controlled conditions applied in Experiment 4 (0.6% agar-gel (20 ml) /10 g. fine /40 g. coarse sand with a food addition similar to 105 mg Tetramin), the test setup can be used for long term monitoring of growth and reproduction with no food limitation or density effects. As food decomposition is reduced with agar-gel, food uptake and the subsequent growth of the worms are more directly the result of addition of food in its original quality.

Regular sediment replacements were performed to prevent bacterial ingrowth and associated food breakdown. Long term stability of the food-agar complex over a period longer than two days has not yet been studied and still remains to be investigated. However, with the regular sediment replacements and the use of agar gel, this standardized setup seems to be most suitable to compare growth and reproduction among an extensive variety of organic waste and food byproducts.

## Conclusions

A novel test for assessing the suitability of food by-products to produce aquatic worm biomass was successfully developed. Growth and reproduction response of Lv can be studied in time with this test. A selected combination of sand, agar gel, and food combined with regular sediment replacements results in less protein decomposition and a higher sediment dwelling activity. The benefit of agar gel application is that ingestion and resulting growth are more directly the result of addition of food in its original quality. This test method is expected to be suitable for a variety of food related studies using other sediment dwelling invertebrates. For waste and byproduct applications from food industries, both organic loading and ammonium should be controlled as high levels influence growth and reproduction of worms in a negative way. To study the bio recovery, this test has his limitations as the current system design does not allow for a reliable quantification of mass fluxes e.g. for N or COD because of their low concentrations and high water renewal rates.

## Supporting Information

S1 Fig Final worm numbers and increase in total wet weight over a three week period for each combination employed in Experiments 2 and 3. Data includes Weekly TAN, Oxygen and pH. Agar concentrations (Conc.) expressed as weight percentage amounts of agar gel and sand in grams, and food in milligrams. Sand fractions used: coarse sand (C) and fine sand (F). Initial worm number = 50 (all tests). Average total start weight Exp. 2: 399 mg (min-max, 330–476), Exp.3: 391 mg (min-max, 350–422). Exp.3, Test 9: Worm losses due to experimental error Weekly TAN, Oxygen and pH for each combination (average ± SD), (n = 3).* loss of approximately 25 worms due to experimental error.
(DOCX)
