# Supplementary material for: Agar Sediment Test for Assessing the Suitability of Organic Waste Streams for Recovering Nutrients by the Aquatic Worm Lumbriculus variegatus
Source: PLoS One. 2016 Mar 3;11(3):e0149165. doi: 10.1371/journal.pone.0149165 (PMC4777450; doi:10.1371/journal.pone.0149165)
Supplement: S1 Fig — Data includes Weekly TAN, Oxygen and pH. Agar concentrations (Conc.) expressed as weight percentage amounts of agar gel and sand in grams, and food in milligrams. Sand fractions used: coarse sand (C) and fine sand (F). Initial worm number = 50 (all tests). Average total start weight Exp. 2: 399 mg (min-max, 330–476), Exp.3: 391 mg (min-max, 350–422). Exp.3, Test 9: Worm losses due to experimental error Weekly TAN, Oxygen and pH for each combination (average ± SD), (n = 3).* loss of approximately 25 worms due to experimental error. (DOCX) [file pone.0149165.s001.docx]

**Supporting Information**

S1, Final worm numbers and increase in total wet weight over a three week period for each combination employed in Experiments 2 and 3 including Weekly TAN, Oxygen and pH. Agar concentrations (Conc.) expressed as weight percentage, amounts of agar gel and sand in grams, and food in milligrams. Sand fractions used: coarse sand (C) and fine sand (F). Initial worm number= 50 (all tests). Average total start weight Exp. 2: 399 mg (min-max, 330-476), Exp.3: 391 mg (min-max, 350-422). Exp.3, Test 9: Worm losses due to experimental error

Weekly TAN, Oxygen and pH for each combination (average ± SD), (n=3).* loss of approximately 25 worms due to experimental error.

|  |  | **Agar** | **Sand** |  | **Final worm** | |  |  |  |
| --- | --- | --- | --- | --- | --- | --- | --- | --- | --- |
| **Exp.** | **Test** | **(concentration) (%)/ Amount)** | **(fraction/amount)** | **Food(amount)** | **Number #** | **T. weight (%)** | **NH_4_-N (mg/L)** | **DO**  **(mg O_2_/L)** | **pH** |
| **2** | 1 | 0 (no addition) | C/90 | 420 | 67 | 9.9 | 1.77 (0.73) | 0.37 (0.10) | 7.31 (0.23) |
|  | 2 |  |  | 1050 | 46 | -12.2 | 1.97 (0.03) | 0.06 (0.02) | 6.81 (0.25) |
|  | 3 |  | F/22.5 + C/67.5 | 420 | 62 | 9.1 | 1.61 (0.35) | 0.08 (0.0*3)* | 7.06 (0.27) |
|  | 4 |  |  | 1050 | 50 | 3.3 | 1.99 (0.58) | 0.05 (0.01) | 7.00 (0.61) |
|  | 5 | 0.6 / 30 | C/90 | 420 | 65 | 41.3 | 0.20 (0.12) | 2.03 (0.48) | 8.11 (0.34) |
|  | 6 |  |  | 1050 | 54 | 7.5 | 1.23 (0.39) | 0.22 (0.15) | 7.06 (0.60) |
|  | 7 |  | F/22.5 + C/67.5 | 420 | 59 | 40.6 | 0.04 (0.01) | 1.24 (1.06) | 7.88 (0.27) |
|  | 8 |  |  | 1050 | 52 | 11.1 | 1.30 (0.31) | 0.05 (0.04) | 7.44 (0.54) |
|  | 9 | 0.8 /30 | C/90 | 420 | 64 | 22.8 | 0.29 (0.11) | 0.26 (0.24) | 7.76 (0.39) |
|  | 10 |  |  | 1050 | 53 | 8.2 | 0.98 (0.25) | 0.14 (0.12) | 7.46 (0.57) |
|  | 11 |  | F/22.5 + C/67.5 | 420 | 76 | 44.5 | 0.15 (0.10) | 0.21 (0.18) | 7.89 (0.25) |
|  | 12 |  |  | 1050 | 54 | 19.1 | 0.69 (0.53) | 0.04 (0.04) | 7.55 (0.40) |
| **3** | 1 | 0 / 0 | F/10 + C/40 | 0 | 104 | -20.6 | 0.23 (0.37) | 8.14 (0.25) | 8.36 (0.07) |
|  | 2 |  |  | 105 | 56 | 3.3 | 0.68 (0.47) | 0.58 (0.63) | 7.27 (0.03) |
|  | 3 |  |  | 140 | 76 | 5.2 | 0.39 (0.14) | 2.00 (2.34) | 7.36 (0.26) |
|  | 4 |  |  | 210 | 50 | -6.1 | 1.17 (1.08) | 0.13 (0.14) | 7.03 (0.11) |
|  | 5 | 0.6 / 20 | F/10 + C/40 | 0 | 97 | -8.8 | 0.02 (0.02) | 3.12 (1.45) | 8.03 (0.26) |
|  | 6 |  |  | 105 | 79 | 38.3 | 0.20 (0.13) | 0.26 (0.23) | 7.39 (0.29) |
|  | 7 |  |  | 140 | 52 | 28.7 | 0.21 (0.09) | 0.10 (0.06) | 7.30 (0.18) |
|  | 8 |  |  | 210 | 50 | -10.8 | 1.22 (1.07) | 0.05 (0.05) | 6.86 (0.22) |
|  | 9 | 0.8 /20 | F/10 + C/40 | 0 | -* | - | 0.05 (0.07) | 1.40 (1.36) | 7.82 (0.20) |
|  | 10 |  |  | 105 | 64 | 22.1 | 0.14 (0.09) | 0.09 (0.01) | 7.27 (0.23) |
|  | 11 |  |  | 140 | 61 | 29.4 | 0.06 (0.03) | 0.11 (0.04) | 7.30 (0.12) |
|  | 12 |  |  | 210 | 50 | 17.5 | 0.55 (0.35) | 0.02 (0.02) | 7.20 (0.17) |
